# Sarcopenia, Obesity, and Sarcopenic Obesity in Relation to Functional Limitations in Older Adults

**DOI:** 10.3390/jcm15031000

**Published:** 2026-01-26

**Authors:** Marika Murawiak, Marta Lewandowicz-Czarnecka, Beata Kaczmarek, Ewa Deskur-Śmielecka, Katarzyna Wieczorowska-Tobis, Roma Krzymińska-Siemaszko

**Affiliations:** 1 Department of Palliative Medicine, Poznan University of Medical Sciences, 61-245 Poznan, Poland; mlewandowicz@ump.edu.pl (M.L.-C.); bkaczmarek@ump.edu.pl (B.K.); edeskur@ump.edu.pl (E.D.-Ś.); tobis@ump.edu.pl (K.W.-T.); krzyminskasiemaszko@ump.edu.pl (R.K.-S.); 2 Doctoral School, Poznan University of Medical Sciences, 60-812 Poznan, Poland

**Keywords:** basic activities of daily living, functional limitations, instrumental activities of daily living, obesity, older adults, sarcopenia, sarcopenic obesity, quality of life

## Abstract

**Background/Objectives**: Sarcopenia, obesity, and sarcopenic obesity (SO) are common in older adults and may be associated with functional limitations in Basic (BADL) and Instrumental (IADL) Activities of Daily Living. This study aimed to evaluate the association between body composition phenotypes and BADL/IADL limitations among older adults. **Methods**: A cross-sectional study included 440 community-dwelling adults aged ≥60 years (281 women, 159 men; mean age 74.7 ± 7.8 years). Sarcopenia was diagnosed according to EWGSOP2 criteria, obesity was defined as Percent Body Fat > 42% in women and >30% in men, and SO was classified based on the ESPEN/EASO recommendations. The reference phenotype was ‘non-sarcopenic, non-obese’. Functional status was evaluated using the Katz and Lawton scales, with limitations defined as BADL ≤ 5 and IADL ≤ 26 points, respectively. Multivariate logistic regression was performed to determine associations between body composition phenotypes and BADL/IADL limitations. **Results**: Over half of the participants (57.1%) had abnormal body composition: 31.6% obesity, 11.4% sarcopenia, and 13.2% SO. Sarcopenic obesity was associated with nearly threefold higher odds of BADL limitations (OR = 2.86; *p* = 0.003) and 3.7-fold higher odds of IADL limitations (OR = 3.68; *p* < 0.001), compared to the reference phenotype. Sarcopenia was associated with IADL limitations only in the unadjusted model (OR = 2.44; *p* = 0.010). Beyond adverse body composition phenotypes, BADL/IADL limitations were also associated with lower muscle strength, multimorbidity, and poorer nutritional status. **Conclusions**: SO was linked to both BADL and IADL limitations, while sarcopenia was associated only with IADL deficits. Isolated obesity showed no consistent relationship with functional impairment. These findings support prioritizing SO in screening and prevention, although the cross-sectional design precludes causal inference.

## 1. Introduction

Aging is probably one of the most important demographic challenges in Europe. In 2024, one out of five citizens (21.6%) of the European Union (EU) was at least 65 years old. It is estimated that this percentage would increase to 32.5% by the year 2100 [1]. This tendency is observed all across Europe, but its rate is highest in Poland [1]. Aging of the population is associated with increased prevalence of functional limitations and disability, defined as the loss of independence in everyday functioning due to physical, cognitive, or sensory impairments [2]. The ability to perform activities of daily living is a key component of successful aging [3,4]. Basic Activities of Daily Living (BADL) refer to elementary aspects of self-care, such as taking meals, dressing, and toileting. In contrast, Instrumental Activities of Daily Living (IADL) refer to more complex activities necessary for functioning in the environment, such as shopping, preparing meals, and housekeeping [2,3,5]. In 2019, nearly half (49.7%) of EU residents aged 65 or older reported moderate to severe problems with activities of daily living [6,7]. The most recent data from 2025 indicate that almost one-third (32.4%) of individuals aged 60 years or older have problems with performing Basic Activities, and as many as 56.9% have problems with Instrumental Activities of Daily Living [2]. Usually, deficits in IADL appear before problems with BADL, and may be an early marker of impairment in functional ability [8,9]. Loss of independence not only decreases quality of life but also increases the risk of institutionalization, hospitalization, and premature death [2,8,10].

Changes in body composition may contribute to functional dependency in older adults [11,12,13]. Both sarcopenia and obesity, first defined as an age-related, progressive loss of muscle mass and strength, and second, as an excessive accumulation of fat tissue, independently increase the risk of mobility limitation and falls [14,15,16,17]. A decline in muscle function is an independent predictor of functional disability, being a stronger predictive factor than low muscle mass itself [11]. Sarcopenic obesity (SO), described as the coexistence of sarcopenia and obesity, is associated with cumulative impairments in musculoskeletal function, leading to functional limitations and dependency [18,19]. Pathophysiology of SO is multifactorial, including hormonal dysregulation, chronic low-grade inflammation (inflammaging), and structural and metabolic changes in muscle and fat tissue [20,21,22]. There is some evidence to indicate that SO may be associated with poorer functional capacity than sarcopenia or obesity alone [23]. However, the quantitative assessment of this relation and the comparison between the results of various studies remain questionable. This is due to substantial heterogeneity in diagnostic criteria and body composition assessment methods, which may affect reported prevalence estimates and functional associations [24]. To address these issues, the European Society for Clinical Nutrition and Metabolism (ESPEN) and the European Association for the Study of Obesity (EASO) proposed a unified definition of SO. They issued diagnostic recommendations for clinical practice in 2022. A unified definition of SO, elaborated by ESPEN/EASO, offers new research options, enabling the assessment of SO prevalence and its clinical consequences [18].

Despite the growing interest in age-related body composition changes, a comprehensive analysis of their impact on functional abilities in older adults remains lacking. Only a limited number of studies have simultaneously compared the three major body composition phenotypes—sarcopenia, obesity, and SO—within a single analytical framework, particularly in relation to limitations in both BADL and IADL [23,25]. Many existing studies have focused on isolated phenotypes, single functional domains, or institutionalized samples. Consequently, the generalizability of their findings to community-dwelling older persons, who represent the fastest-growing segment of the elderly population, is limited [26,27,28]. Improved understanding of the relationships between different body composition phenotypes and limitations in BADL/IADL may facilitate the early identification of individuals at risk of losing functional capacity. Moreover, such insights may contribute to the development of effective preventive and rehabilitative strategies in aging populations [29,30]. The aim of the present study was to analyze the associations between body composition phenotypes and the ability to perform BADL and IADL in the community-dwelling older adults in Poland. To the best of our knowledge, this study is among the first conducted in Central-Eastern Europe to provide a comprehensive assessment of the relationship between the three body composition phenotypes and everyday functioning of older adults living in the community.

## 2. Materials and Methods

### 2.1. Study Sample

This cross-sectional study included 440 individuals aged 60 years or older, living independently in the community in Poland. Participants were recruited from senior community centers, local Universities of the Third Age, and primary care clinics. Data were collected from September 2023 to June 2025. All assessments were conducted by trained healthcare professionals with experience in geriatric research. The evaluators had a background in health sciences (including dietetics and physiotherapy) and completed standardized training prior to data collection. The training covered cognitive screening using the AMTS, body composition measurements, functional performance tests, and nutritional assessment, thereby ensuring methodological consistency and minimizing inter-observer variability.

Exclusion criteria included cognitive impairment and contraindications to body composition analysis using the bioimpedance method (BIA). These included implantable devices (e.g., cardiac pacemakers, cardioverter-defibrillators), metal implants, significant edema, and the inability to maintain a standing position required for anthropometric and body composition measurements.

Cognitive function was assessed with a 10-item Abbreviated Mental Test Score (AMTS). A score of ≥7 indicated uncompromised cognitive function required for study enrollment [31].

All participants gave informed consent to the study. The Bioethics Committee of the Poznan University of Medical Sciences accepted the study protocol (number of approval: 459/24).

### 2.2. Assessment of Body Composition Phenotypes

Body composition analysis. Body composition was analyzed using a BIA method and an InBody 120 analyzer (Biospace, Seoul, South Korea). The BIA method is based on measurements of tissue resistance and reactance in response to a low-intensity electric current. It enables fast and non-invasive assessment of body composition parameters, such as body weight (W), Body Mass Index (BMI), Skeletal Muscle Mass (SMM), Percent Body Fat (PBF), fat mass (FM), and segmental lean body mass of trunk and limbs [32].

Sarcopenia phenotype. Sarcopenia was diagnosed based on criteria suggested in 2018 by the European Working Group on Sarcopenia in Older People 2 (EWGSOP2) [14]. Because a complete assessment of body composition, including both muscle mass and muscle strength, was performed in all participants, the screening stage with the Strength, Assistance with walking, Rising from a chair, Climbing stairs, and Falls (SARC-F) questionnaire was omitted. This approach did not compromise diagnostic validity, as all participants were assessed using objective EWGSOP2 measures. According to the EWGSOP2 algorithm, sarcopenia was considered probable in cases of decreased muscle strength in the upper and/or lower limbs. Sarcopenia was confirmed by concomitant low muscle mass, assessed with BIA parameters [14].

- Upper limb muscle strength was assessed with a Hand Grip Strength test (HGS), using a hand dynamometer (Saehan, Changwon, Korea). The measurements were performed in a sitting position, with shoulders adducted and elbows flexed at 90°, twice for each hand. The best result out of four taken was compared with diagnostic thresholds. Cut-off points for low muscle strength were <16 kg in women and <27 kg in men [14];
- Lower limb muscle strength was assessed with a Five-Repetition Sit-to-Stand test (5STS). Participants were seated in a chair without armrests, with their arms crossed at their chest. They were instructed to stand up and sit down five times at the given sign as quickly as possible, without using their hands. Test times longer than 15 s indicated reduced lower limb muscle strength [14];
- Muscle mass was assessed based on the Appendicular Lean Mass Index (ALM Index), defined as the sum of lean mass of lower and upper limbs divided by squared height (kg/m^2^) [13]. Low muscle mass was defined using cut-off points specific to the Polish population: <5.6 kg/m^2^ for women and <7.4 kg/m^2^ for men [33].

Obesity phenotype. Obesity phenotype was diagnosed with PBF parameter and the following cut-off points: >42% for women, >30% for men [34]. This approach ensured methodological consistency with the ESPEN/EASO recommendations using PBF thresholds as diagnostic criteria for sarcopenic obesity. As a result, direct comparison between obesity and sarcopenic obesity phenotypes were possible [18].

Sarcopenic obesity phenotype. SO phenotype was defined by the ESPEN/EASO diagnostic algorithm, with the screening phase omitted [18]. Participants were classified as having SO phenotype if they had concomitant:

- Low muscle strength defined as HGS < 16 kg in women and <27 kg in men, and/or 5STS > 15 s [18,35];
- Low muscle mass based on percentage of Skeletal Muscle Mass in total body mass (SMM/W). Cut-off points were <27.6% in women and <37.0% in men [18,36];
- Excessive percentage of fat tissue, defined as PBF > 42% in women and >30% in men [18,34].

Based on the above criteria, all participants were classified into one of four phenotype groups: sarcopenia without obesity, obesity without sarcopenia, sarcopenic obesity, or non-sarcopenic, non-obese phenotype.

### 2.3. Functional Capacity

Basic Activities of Daily Living (BADL). Basic Activities of Daily Living were assessed using the Katz scale, which includes six areas: bathing, dressing, toileting, transferring (in and out of bed or chair), continence, and taking meals [37]. Each area was scored as follows: 1—fully independent, 0.5—requires some help, 0—entirely dependent. Maximum score—6 points—indicated complete functional independence. Consistent with previous studies, BADL scores were dichotomized into no limitation versus at least one limitation. A score of ≤5 points indicated limited independence in BADL [38,39,40,41,42,43]. This approach allowed the exclusion of participants whose only deficit was urinary incontinence, as this condition alone was not considered indicative of functional limitation.

Instrumental Activities of Daily Living (IADL). Ability to perform IADL was assessed with the Lawton scale, comprising nine items: using a telephone, shopping, food preparation, housekeeping, home repairs, laundry, mode of transportation, responsibility for taking medications, and ability to handle finances [44]. Each item was scored 1–3 (3—fully independent, 2—partially dependent, 1—dependent). The maximum score was 27. In line with previous studies, IADL scores were classified as indicating either no limitation or at least one limitation. A score of ≤26 points indicated limited independence in IADL [38,39,40,41,42,43].

### 2.4. Nutritional Status

Nutritional status was assessed using the Mini Nutritional Assessment (MNA) questionnaire. In this study, the full version of the MNA (MNA-full) was administered to all participants, regardless of their MNA-Short Form (MNA-SF) screening score. The MNA-full questionnaire consists of 18 items. These items cover anthropometric variables, qualitative and quantitative assessment of the diet, mode of feeding and living, and self-assessment of nutritional status and health condition. The total score was classified as follows: ≥24—normal nutritional status, 17–23.5—at risk of malnutrition, <17—malnutrition [45]. All participants scoring fewer than 24 points were pooled into a single category—poor nutritional status (PNS).

### 2.5. Concomitant Variables

Health status n was characterized by the number of chronic diseases, based on medical history and documentation, and the number of prescription drugs taken on a regular basis.

### 2.6. Sample Size Calculation

The sample size was calculated using G*Power 3.1.9.7 (Heinrich-Heine-Universität Düsseldorf, Düsseldorf, Germany). The effect size used in the calculation was derived from the study by Bahat et al. [46]. Based on the reported prevalence of SO associated with IADL impairment, assuming a type I error rate of 0.05 and a statistical power of 80%, the minimum required sample size was estimated to be 345 participants.

### 2.7. Statistical Analysis

Statistical analyses were performed with STATISTICA 10 PL (Statsoft, Krakow, Poland) and PQStat 1.8.6 (PQStat Software, Poznan, Poland). Categorical variables were presented as numbers (n) and percentages (%), while quantitative variables were presented as mean ± standard deviation (SD). Between-group comparisons were performed using the Student *t*-test or the Cochrane-Cox test, depending on the homogeneity of variance. Comparisons between three or more groups (sarcopenia, obesity, SO, and non-sarcopenic, non-obese phenotype) were performed with analysis of variance ANOVA or Welch F test, depending on the homogeneity of variance, and Bonferroni test as a post hoc procedure. Homogeneity of variance was assessed with the Levene test. Correlations between quantitative variables were assessed with the Spearman correlation coefficient. Univariate and multivariate logistic regression models were used to verify the association between selected factors and functional limitations in BADL (Katz score ≤ 5) and IADL (Lawton score ≤ 26). Qualitative variables were assessed with Pearson’s chi-square test or likelihood-ratio chi-square in case of low expected frequencies in the contingency table’s cells. *p* value < 0.05 was considered significant.

## 3. Results

### 3.1. General Characteristics of the Study Sample

Data on 440 persons aged 60 years and older (mean age 74.7 ± 7.8, 63.9% of women) were collected. One out of four participants had low muscle mass by the ALM index. The prevalence of low muscle mass was twofold higher in men as compared to women (34.6% vs. 19.6%; *p* < 0.001). Half of the study sample (50.0%) fulfilled the EWGSOP2 criteria for probable sarcopenia based on low muscle strength parameters. Reduced muscle strength was more frequently observed in the lower limbs than in the upper limbs (38.2% vs. 27.5%, respectively). Poor nutritional status was found in 36.8% participants, including 3.2% malnourished persons and 33.6% at risk of malnutrition. Persons included in the study had four chronic diseases on average (4.3 ± 2.5) and were taking more than six medications daily (6.4 ± 3.9). Despite relatively high mean functional capacity scores (BADL 5.5 ± 0.7; IADL 23.9 ± 4.4), more than half of the study participants (55.9%) reported difficulty with at least one Instrumental Activity of Daily Living. In addition, one in five persons (20.9%) had problems with BADL. These results did not differ between sexes. Detailed data (by sex) are shown in Table 1.

### 3.2. Prevalence of Body Composition Phenotypes

More than half of the study sample (57.1%) had abnormal body composition: 31.6% participants were obese, 11.4% had sarcopenia, and 13.2% had sarcopenic obesity. The prevalence of abnormal body composition was higher in men than in women (62.9% vs. 52.3%, respectively; *p* = 0.017). The difference was particularly evident for sarcopenic obesity (19.5% in men vs. 9.6% in women; *p* = 0.003). Detailed data (by sex) are shown in Table 2.

### 3.3. Body Composition Phenotypes—Characteristics of Phenotype Groups

Sarcopenia and SO were associated with less favorable clinical profiles and worse nutritional status in comparison with obesity and non-sarcopenic, non-obese phenotype. Compared with all other phenotype groups, participants with sarcopenia were the oldest (77.5 ± 8.4 years; *p* < 0.001 vs. obesity). They also had the lowest body weight (55.2 ± 9.4 kg; *p* < 0.001), BMI (21.7 ± 2.8 kg/m^2^; *p* < 0.001), SMM (21.0 ± 4.2 kg; *p* < 0.001 vs. obesity), and FFM (39.2 ± 7.2 kg; *p* < 0.001 vs. obesity). Health burden was higher in the sarcopenia group (4.4 ± 2.2 diseases; 6.5 ± 2.6 medications) than in persons with non-sarcopenic, non-obese phenotype. However, it was lower than in the SO group, which showed the highest level of multimorbidity (5.9 ± 2.5; *p* < 0.001) and polypharmacy (9.1 ± 4.4; *p* < 0.001). Among all phenotype groups, participants with SO had the highest body weight (87.5 ± 14.1 kg), BMI (33.8 ± 5.7 kg/m^2^), and PBF (43.4 ± 7.2%), all of which were significantly higher than in both the reference and sarcopenia groups (all *p* < 0.001). Individuals with obesity were the youngest (72.9 ± 7.6 years) and had the best muscle profile. Compared with the SO group, they had a lower number of chronic conditions (4.3 ± 2.6) and took fewer medications (6.6 ± 3.8) (*p* < 0.001 for both), with levels similar to those observed in the sarcopenia group. Comprehensive characteristics of body composition phenotype groups are shown in Table 3.

### 3.4. Body Composition Phenotypes—Functional Capacity BADL/IADL

One-way analysis of variance (ANOVA) revealed significant differences between phenotype groups in the ability to perform both basic and Instrumental Activities of Daily Living (*p* < 0.001). Individuals with sarcopenic obesity had the worst functional profile. The proportion of participants with limitations in BADL was twofold higher than in other phenotype groups (41.4%; *p* < 0.001), and the vast majority had problems with IADL (81.0%; *p* < 0.001). The mean BADL and IADL scores were the lowest in participants with SO (5.1 ± 0.9; *p* < 0.001, and 20.9 ± 5.4, respectively). These scores were significantly lower than those observed in the non-sarcopenic, non-obese and obesity groups (both *p* < 0.001), but did not differ significantly from the sarcopenia group (*p* = 0.053). While the prevalence of BADL limitations was comparable across the sarcopenia, obesity, and non-sarcopenic, non-obese phenotype groups (approximately 20.0%), deficits in IADL were significantly more frequent in individuals with sarcopenia than in those with obesity (72.0% vs. 45.3%; *p* < 0.001). The mean IADL score was also lower in the group with sarcopenia in comparison with the one with obesity (23.0 ± 3.9 vs. 25.0 ± 3.6; *p* = 0.024).

**Figure 1:**
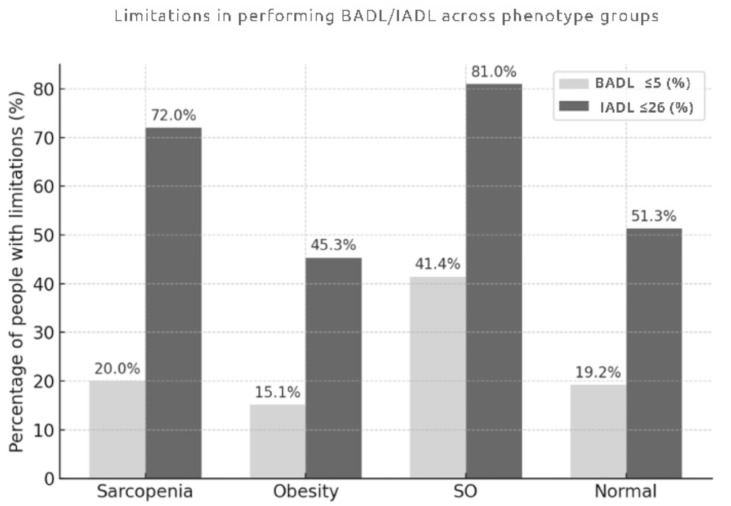
Limitations in performing BADL/IADL across phenotype groups. Abbreviations: BADL, Basic Activities of Daily Living; IADL, Instrumental Activities of Daily Living; SO, sarcopenic obesity.

Participants with obesity had the most favorable functional profile of all four body composition phenotype groups. Only 15.1% of the obese individuals had problems with performing Basic Activities of Daily Living; this was the lowest prevalence among the study groups and was significantly lower than that observed in the SO group (*p* < 0.001). The percentage of obese people with deficits in IADL was 45.3% and was significantly lower than in the sarcopenia group (72.0%; *p* < 0.001) and SO group (81.0%; *p* < 0.001). Among the four phenotype groups, mean scores for BADL (5.6 ± 0.7) and IADL (25.0 ± 3.6) were the highest in the obesity one. Individuals with non-sarcopenic, non-obese phenotype had a moderate level of functional ability: 19.2% of them had limitations in performing BADL, and 51.3% in IADL. Both percentages were significantly lower than those observed in the SO group (*p* < 0.001) and were comparable to the values found in sarcopenia and obesity groups. In the non-sarcopenic, non-obese phenotype group, mean BADL and IADL scores (5.6 ± 0.7 and 24.2 ± 4.3, respectively) were similar to those in the obesity group and significantly higher than in the SO group (*p* < 0.001). Detailed data are shown in Table 3 and Figure 1.

### 3.5. Relationship Between Age, Sex, Body Composition Phenotypes, and Functional Disability

In women, the four phenotype groups had comparable prevalence of limitations in BADL (*p* = 0.237) but differed in IADL (*p* = 0.012). Full ability to perform Instrumental Activities of Daily Living was the most common in women with non-sarcopenic, non-obese phenotype (50.7%), and the least common in SO (18.5%). Among men, BADL limitations were most common in the SO group (45.2%; *p* < 0.001). Limitations in IADL occurred most frequently in the individuals with sarcopenia (83.3%; *p* < 0.001). The lowest prevalence of limited functional capacity was found in the obesity group (ADL 5.9%; IADL 31.4%). Detailed data are presented in Table 4.

### 3.6. Factors Associated with Limitations in BADL and IADL

Multivariable logistic regression analysis showed significant associations between some clinical variables and functional limitations in Basic and Instrumental Activities of Daily Living. The number of chronic diseases was associated with BADL disability, and each additional disease increased the odds of BADL limitations by 22.5% (OR = 1.225; *p* = 0.003). Nutritional status had protective effects: an increase in the MNA score by 1 reduced the likelihood of deficits in BADL by 15.8% (OR = 0.842; *p* < 0.001). Reduced lower limb muscle strength was the strongest factor associated with functional limitations. Persons with reduced lower limb muscle strength had approximately threefold higher odds of BADL difficulties (OR = 3.225; *p* < 0.001). Other variables, such as number of medications, reduced upper limb muscle mass, low ALM Index, BMI, and PBF, had no association with limitations in BADL.

Reduced lower limb muscle strength also had the strongest association with limitations in IADL. The odds was fourfold higher in individuals with reduced strength (OR = 4.297; *p* < 0.001). Unlike for BADL, low upper limb muscle strength increased the odds of deficits in IADL twofold (OR = 2.261; *p* = 0.011). Nutritional status was another important factor associated with IADL disability. Each additional point in the MNA score reduced the likelihood by 13.4% (OR = 0.866; *p* = 0.001). The likelihood of IADL limitations was increased by 20.8% (OR = 1.208; *p* = 0.009) with each chronic disease and by 9.9% (OR = 1.099; *p* = 0.024) with each medication. The odds of difficulties with performing IADL also increased with BMI (OR = 1.107; *p* = 0.024). Detailed data are shown in Table 5.

The association between body composition phenotypes and BADL/IADL limitations was examined with a separate regression analysis (Table 6). After adjustment for sex and age individuals with SO phenotype had nearly threefold higher odds of BADL limitations (OR = 2.859; *p* = 0.003) and more than threefold higher odds of IADL limitations (OR = 3.675; *p* < 0.001) compared to participants in the reference phenotype group. Sarcopenia was associated with IADL deficits in the unadjusted model (OR = 2.442; *p* = 0.010); however, this association was attenuated and no longer statistically significant after adjustment for sex and age (*p* = 0.064). Obesity phenotype was not associated with functional limitations. Detailed data are shown in Table 6.

## 4. Discussion

To the best of our knowledge, this is the first study conducted in Central-Eastern Europe assessing the relation between functional disability and sarcopenia, obesity, and sarcopenic obesity in community-dwelling older adults, using the ESPEN/EASO guidelines for the diagnosis of sarcopenic obesity. As many as 57.1% of our study population had abnormal body composition phenotype. Obesity was the most prevalent one (31.6%), followed by SO (13.2%) and sarcopenia (11.4%). Sarcopenic obesity showed the strongest, independent association with functional disability. Participants with the SO phenotype had approximately 3-fold higher odds of limitations in Basic Activities of Daily Living and 3.5-fold higher odds of limitations in Instrumental Activities of Daily Living compared with those with the non-sarcopenic, non-obese phenotype. Sarcopenia was associated with limitations in the ability to perform IADL only in the unadjusted model (OR = 2.5). Obesity was not related to functional capacity in our study sample.

To date, the relationship between four body composition phenotypes and the risk of disability in performing BADL and IADL has been largely unexplored. Only one relevant study was identified, conducted by Bahat et al. [46]. This study included 1468 elderly persons living in the Turkish community (68.8% of women; median age 75 years). The prevalence of pathological phenotypes in their study differed from that in our sample (45.4% participants with obesity, 3.7% with sarcopenia, and 3.7% with SO phenotype). It should be noted that Bahat et al. [46] used a different methodological approach to assess body composition phenotypes. Specifically, the SO phenotype was diagnosed based on LMM-to-BMI ratio rather than the SMM-to-body weight proportion recommended by the EASO/ESPEN consensus and applied in the present study. Despite the various prevalences of particular body phenotypes, SO was consistently associated with disability in activities of daily living in both studies. Moreover, the strength of this association was comparable with Bahat et al. [46] reporting odds ratios of 2.7 for BADL and IADL, compared with odds ratios of 3.0 for BADL (*p* = 0.001) and 3.5 for IADL (*p* = 0.002) in our study. In contrast to our findings, Bahat et al. [46] observed a stronger association for sarcopenia with odds ratios of 3.4 for BADL (*p* < 0.001) and 6.4 for IADL (*p* < 0.001). In our sample, sarcopenia was associated only with limitations in the ability to perform IADL, and this relationship was observed exclusively in the unadjusted model (OR = 2.4, *p* = 0.010). Unlike in the present study, obesity was associated with functional decline in both BADL and IADL in the study by Bahat et al. [46], although the magnitude of the effect was modest (OR = 1.5 and OR = 1.3, respectively).

The observed associations between body composition phenotypes and functional limitations are biologically reasonable and likely result from different, yet partially similar, pathophysiological mechanisms. In sarcopenia, age-related neuromuscular alterations, impaired anabolic responsiveness, and hormonal changes contribute to declines in muscle mass and strength, leading to reduced physical performance [11,47,48,49]. In obesity, functional capacity may be limited by increased mechanical load, altered biomechanics, and metabolic dysregulation (e.g., insulin resistance and chronic low-grade inflammation), which can impair muscle quality despite relatively preserved muscle mass [12,13,15,16,17]. In SO, expansion of visceral adipose tissue plays a central role in its pathogenesis through increased secretion of proinflammatory cytokines (e.g., TNF-α, IL-6), perpetuation of chronic low-grade inflammation, and the development of insulin resistance. These processes contribute to muscle lipotoxicity, mitochondrial dysfunction, impaired muscle quality, and anabolic resistance. This pathophysiological profile is consistent with the concept of “inflammaging,” whereby impaired adipose–muscle interactions drive metabolic dysregulation. Common geriatric burdens, including multimorbidity, polypharmacy, and poor nutritional status, further exacerbate sarcopenic obesity, accelerating functional decline and disability risk [18,19,20,21,22,23]. Our findings suggests that obesity concomitant with loss of muscle mass and strength is a marker of cumulated health burden. The German study KORA-Age, including 998 elderly persons (mean age 75.6 years), yielded similar results: SO phenotype was associated with increased risk of multimorbidity (OR = 2.59), polypharmacy (OR = 1.96), and cognitive dysfunction (OR = 3.03), independent of confounding factors [50]. Multimorbidity and polypharmacy were independently associated with decreased functional capacity in our research, as well as in some other studies. For example, in cross-sectional analysis by Tachall et al. [51], polypharmacy was associated with increased risk of limitations in BADL (OR = 1.87) and IADL (OR = 3.52), regardless of age and other clinical variables. Ćwirlej-Sozańska et al. [52] observed a higher risk of functional limitations in persons taking ≥4 medications; each chronic condition increased the risk of IADL limitations by 18%.

In the present study, SO was observed more than twice as often in men as in women (19.5% vs. 9.6%). Similarly, in the FIBRA-RJ study, which included 270 community-dwelling older adults from Brazil (189 women and 81 men; mean age 77.5 ± 5.9 years). Sarcopenic obesity was identified in 29.3% of participants and was more than four times as prevalent in men than in women (63.0% vs. 14.7%) [53]. The higher occurrence of SO in men may be explained by sex-specific differences in body fat distribution and lifestyle changes associated with aging. Men are more prone to accumulate visceral adipose tissue, which exerts stronger metabolic and proinflammatory effects and may accelerate muscle loss, thereby promoting the development of the SO phenotype [54,55,56]. Moreover, the marked reduction in physical activity, commonly observed in men after retirement, may further exacerbate muscle decline, while fat mass remains stable or even increases [57].

The broader limitations observed in individuals with SO across both BADL and IADL likely reflect a reduced functional reserve compared with isolated phenotypes. While isolated sarcopenia may initially impair strength and coordination-dependent tasks, typically manifesting as difficulties in more complex IADL, sarcopenic obesity appears to result in earlier and more profound impairment, extending to BADL. These results are perfectly in line with the consensus review by the Global Leadership Initiative in Sarcopenia (GLIS) [58]. The GLIS experts stated that although sarcopenia may have a moderate association with disability in IADL, the data about its relationship with BADL are inconsistent and significantly vary between studies [58]. They emphasized that the ability to perform Instrumental Activities of Daily Living may be a more sensitive index of functional consequences of sarcopenia. This is because IADL have a more complex character, requiring not only physical capacity, but also a good level of coordination, executive functions, and cognitive skills [58]. Although the present analysis focused on body composition phenotypes, it should be noted that the key components of sarcopenia—reduced lower and upper limb muscle strength—were independent and strongly risk-associated with functional disability. Reduced lower limb muscle strength was associated with a threefold increase in BADL limitations and a fourfold increase in IADL limitations. Reduced upper limb muscle strength was associated with a twofold increase in IADL disability) [58].

Participants with an obesity phenotype had relatively good muscle strength and functional capacity, comparable to those observed in subjects with a non-sarcopenic non-obese phenotype. These findings suggest that moderate excess in fat tissue does not necessarily lead to functional impairment, provided it is not accompanied by a reduction in muscle mass and strength. Therefore, the assessment of functional disability in older persons should be based on body composition, defined as the proportion of fat and muscle components, and not on anthropometric parameters (e.g., BMI) alone. Our findings add to the “obesity paradox”—a conception that a moderate excess in body mass in elderly persons may serve as a metabolic reserve and may be associated with a favorable prognosis [59,60]. This hypothesis has recently been supported by the results of a study performed in New Zealand, which included nearly 200,000 institutionalized older adults. Obese and overweight persons (BMI 25.0–34.9 kg/m^2^) had better abilities to perform Activities of Daily Living than individuals with normal weight, while underweight (BMI < 18.5 kg/m^2^) was strongly associated with functional disability [61]. Notably, the obesity paradox remains debated, as observational evidence is vulnerable to residual confounding and reverse causation.

Participants with non-sarcopenic, non-obese phenotype had the best functional and health status among all phenotype groups. We use the term ‘non-sarcopenic, non-obese phenotype’ instead of ‘healthy phenotype’ to emphasize its equivocal clinical profile. Some individuals with non-sarcopenic, non-obese phenotype had decreased muscle mass or strength, which may indicate a preclinical phase of sarcopenia. The number of chronic diseases and medications taken on a regular basis in this group were comparable to those found in persons with sarcopenia. These observations may indicate that not all people with non-sarcopenic, non-obese phenotype can be classified as healthy aging. Many of them may be subject to normal aging, and multimorbidity and polypharmacy may occur in them independently of body composition or functional capacity.

Our study has some limitations. Firstly, the cross-sectional character of analysis makes it impossible to draw any conclusions on a possible causal relationship. The directionality of the observed associations cannot be established, and reverse causation cannot be excluded. Second, body composition was assessed using the bioimpedance method. BIA-derived estimates of muscle mass and adiposity are sensitive to hydration status, disease-related fluid shifts, and short-term biological variability, particularly in older and obese individuals. In these populations, BIA may underestimate body fat and overestimate fat-free and Skeletal Muscle Mass. This may result in misclassification of body composition phenotypes, including sarcopenic obesity [62,63,64]. Although major demographic and clinical factors were considered, detailed data on objectively measured physical activity and dietary intake were not available and could not be included in the analyses. This may have partly influenced the observed associations, as physical activity and diet are key determinants of both body composition phenotypes and functional performance. In addition, while participants with overt cognitive impairment were excluded based on AMTS screening, this instrument does not capture subtle or domain-specific cognitive deficits that may still affect functional abilities and limitations in daily activities. Finally, the study included community-dwelling older adults recruited within a specific national and healthcare context, which may limit generalizability to institutionalized populations and other settings.

Despite these limitations, the study has several strengths, including a relatively large and well-characterized sample and comprehensive assessment of body composition, muscle strength, nutritional status, and functional outcomes. The study sample consisted of community-dwelling older persons, which gives better insight into the functional capacity of this population, being an important target for social policy. The phenotype-based classification enabled differentiation between sarcopenia, obesity, and SO, providing clinically relevant insight into their distinct functional profiles. Classification of body composition phenotypes was based on the most recent diagnostic criteria, including the definition of SO according to ESPEN/EASO recommendations. Importantly, phenotype definitions were strengthened by the use of different muscle mass indicators for sarcopenia and sarcopenic obesity. Sarcopenia was defined using the ALM index, reflecting an absolute deficit in muscle mass, whereas SO was characterized using relative muscle mass indices adjusted for body weight, which more accurately capture muscle inadequacy in the context of excess adiposity. In obese individuals, height-adjusted muscle mass indices may underestimate muscle impairment, as increased body weight can mask muscle deficiency. Although this approach may affect prevalence estimates, it aligns with current expert recommendations, enhances validity and clinical relevance, and enables a more accurate characterization of sarcopenia and SO as related but distinct conditions.

## 5. Future Perspectives

Future research should build on the present findings using longitudinal designs to clarify temporal relationships between sarcopenia, SO, and functional decline. The use of reference methods for body composition assessment, such as DXA, with repeated measurements would enable more precise tracking of changes in muscle and fat mass and reduce phenotype misclassification. Including additional factors, such as obesity severity, fat distribution, physical performance, and objectively measured physical activity, may further improve risk stratification. In parallel, intervention studies targeting sarcopenic obesity and the development of feasible screening tools for routine clinical practice are needed to facilitate early identification and targeted management.

By investigating the relationship between body composition phenotypes and functional ability in BADL and IADL, this study provides evidence with direct relevance for clinical assessment, prevention, and rehabilitation in aging populations. These results highlight the importance of early identification of adverse body composition profiles using assessments that go beyond BMI alone. Management should focus on improving body composition quality rather than weight reduction per se. Particular emphasis should be put on preserving or increasing muscle mass and strength while reducing excess adiposity. Multicomponent strategies combining resistance training with adequate protein and energy intake appear especially relevant for SO and may also benefit individuals with sarcopenia. While causal inference is not possible, the findings support early, targeted geriatric approaches to help maintain functional independence. Taken together, these results support implementing routine body composition screening in geriatric care.

## 6. Conclusions

Sarcopenic obesity was associated with limitations in both BADL and IADL, whereas isolated sarcopenia showed a more restricted association with IADL only. This pattern suggests that the coexistence of muscle impairment and excess adiposity constitutes a particularly adverse phenotype for functional status. In contrast, isolated obesity was not consistently associated with functional limitations. This finding underscores the central role of muscle impairment in functional decline and highlights sarcopenic obesity as a priority target for screening and prevention strategies aimed at preserving functional independence in older adults. Nevertheless, given the cross-sectional design and methodological limitations, these findings should be interpreted as descriptive rather than causal. Longitudinal studies are needed to clarify the temporal relationships and mechanisms linking body composition phenotypes with everyday functioning in older adults.

## Figures and Tables

**Table 1:** Characteristics of study sample by sex.

Variable	Women *n* = 281	Men *n* = 159	*p* Value
	Mean ± SD	Mean ± SD	
Age [years]	74.84 ± 7.66	74.49 ± 8.14	0.658
Body weight [kg]	70.40 ± 15.38	79.65 ± 14.87	**<0.001**
BMI [kg/m^2^]	28.81 ± 6.20	27.44 ± 4.47	**0.008**
PBF [%]	38.92 ± 8.92	29.60 ± 7.64	**<0.001**
SMM [kg]	22.46 ± 3.34	30.67 ± 4.71	**<0.001**
FFM [kg]	41.78 ± 5.79	55.35 ± 7.64	**<0.001**
ALM index [kg/m^2^]	6.55 ± 1.03	7.85 ± 0.96	**<0.001**
SMM/W [%]	0.33 ± 0.05	0.39 ± 0.05	**<0.001**
HGS [kg]	20.39 ± 5.43	32.77 ± 9.47	**<0.001**
5STS [s]	14.22 ± 6.95	14.30 ± 7.90	0.915
MNA score	24.39 ± 3.58	24.57 ± 3.36	0.598
Number of chronic diseases	4.49 ± 2.54	3.91 ± 2.24	**0.013**
Number of medications	6.18 ± 3.96	6.77 ± 3.75	0.121
BADL score	5.50 ± 0.67	5.59 ± 0.73	0.202
IADL score	24.12 ± 4.11	24.60 ± 3.35	0.185
	*n* (%)	*n* (%)	
Low muscle mass (ALM Index)	55 (19.6)	55 (34.6)	**<0.001**
Low muscle mass (SMM/W)	40 (14.2)	55 (34.6)	**<0.001**
Low upper limb strength	73 (26.0)	47 (29.6)	0.485
Low lower limb strength	113 (40.2)	55 (34.6)	0.287
Probable sarcopenia	145 (51.6)	75 (47.2)	0.427
Limitations in BADL ≤ 5	61 (21.7)	31 (19.5)	0.584
Limitations in IADL ≤ 26	156 (55.5)	89 (56.0)	0.926
Poor nutritional status	102 (36.3)	60 (37.8)	0.844
MNA			
Malnutrition	10 (3.6)	4 (2.5)	0.752
At risk of malnutrition	92 (32.7)	56 (35.2)	0.672
Normal nutritional status	179 (63.7)	99 (62.3)	0.844

Notes: Quantitative variables shown as mean ± standard deviation (SD), categorical variables as number (*n*) and percentage (%). **Bold** indicates statistically significant results (*p* < 0.05). Abbreviations: BMI, Body Mass Index; PBF, Percent Body Fat; SMM, Skeletal Muscle Mass; FFM, Fat-Free Mass; ALM Index, Appendicular Lean Mass Index; SMM/W, Skeletal Muscle Mass to Weight Ratio; HGS, Hand Grip Strength; 5STS, Five Times Sit-to-Stand Test; MNA, Mini Nutritional Assessment (full version); BADL, Basic Activities of Daily Living; IADL, Instrumental Activities of Daily Living.

**Table 2:** Body composition phenotypes by sex.

Variable	Women *n* = 281	Men *n* = 159	
	*n* (%)	*n* (%)	*p* Value
Body composition phenotypes			**0.017**
Sarcopenia	32 (11.4)	18 (11.3)	0.983
Obesity	88 (31.3)	51 (31.1)	0.869
Sarcopenic obesity	27 (9.6)	31 (19.5)	**0.003**
Non-sarcopenic, non-obese	134 (47.7)	59 (37.1)	**0.032**

Notes: Categorical variables as number (*n*) and percentage (%). **Bold** indicates statistically significant results (*p* < 0.05).

**Table 3:** Characteristics of phenotype groups.

Variable	Body Composition Phenotypes				*p* Value
	Sarcopenia *n* = 50	Obesity *n* = 139	SO *n* = 58	Non-Sarcopenic Non-Obese *n* = 193	
	Mean ± SD	Mean ± SD	Mean ± SD	Mean ± SD	
Age [years]	77.52 ± 8.43	72.91 ± 7.64	77.09 ± 7.39	74.58 ± 7.60	**<0.001**
Body weight [kg]	55.19 ± 9.39	84.14 ± 11.1	87.51 ± 14.10	66.93 ± 11.36	**<0.001**
BMI [kg/m^2^]	21.74 ± 2.76	32.30 ± 3.46	33.78 ± 5.72	25.52 ± 3.68	**<0.001**
PBF [%]	27.74 ± 7.87	41.68 ± 6.18	43.40 ± 7.15	30.81 ± 8.20	**<0.001**
SMM [kg]	20.95 ± 4.19	27.15 ± 5.72	26.60 ± 5.10	25.00 ± 5.14	**<0.001**
FFM [kg]	39.19 ± 7.21	49.13 ± 8.92	49.41 ± 8.63	46.04 ± 8.94	**<0.001**
ALM index [kg/m^2^]	5.76 ± 0.87	7.51 ± 1.02	7.50 ± 1.02	6.85 ± 1.11	**<0.001**
SMM/W [%]	0.38 ± 0.04	0.32 ± 0.05	0.31 ± 0.04	0.37 ± 0.05	**<0.001**
HGS [kg]	18.57 ± 4.89	27.76 ± 9.97	21.88 ± 8.48	25.31 ± 8.93	**<0.001**
5STS [s]	15.79 ± 6.96	13.09 ± 6.09	18.43 ± 9.45	13.42 ± 6.99	**<0.001**
MNA score	21.71 ± 4.19	25.58 ± 2.90	24.01 ± 2.22	24.52 ± 3.62	**<0.001**
Number of chronic diseases	4.44 ± 2.21	4.25 ± 2.55	5.90 ± 2.50	3.77 ± 2.20	**<0.001**
Number of medications	6.50 ± 2.57	6.62 ± 3.76	9.05 ± 4.38	5.41 ± 3.68	**<0.001**
BADL score	5.59 ± 0.40	5.63 ± 0.69	5.14 ± 0.86	5.57 ± 0.67	**0.002**
IADL score	23.00 ± 3.85	24.99 ± 3.55	20.88 ± 5.42	24.20 ± 4.26	**<0.001**
	*n* (%)	*n* (%)	*n* (%)	*n* (%)	
Sex					
Women	32 (64.0)	88 (63.3)	27 (46.6)	134 (69.4)	**0.017**
Men	18 (36.0)	51 (36.7)	31 (53.4)	59 (30.6)	
Low muscle mass (ALM Index)	50 (100.0)	8 (5.8)	18 (31.0)	34 (17.6)	**<0.001**
Low muscle mass (SMM/W)	0 (0.0)	36 (25.9)	58 (100.0)	1 (0.5)	**<0.001**
Low upper limb strength	40 (80.0)	17 (12.2)	31 (53.4)	32 (16.6)	**<0.001**
Low lower limb strength	29 (58.0)	36 (25.9)	46 (79.3)	57 (29.5)	**<0.001**
Probable sarcopenia	50 (100.0)	45 (32.4)	58 (100.0)	67 (34.7)	**<0.001**
Limitations in BADL ≤ 5	10 (20.0)	21 (15.1)	24 (41.4)	37 (19.2)	**<0.001**
Limitations in IADL ≤ 26	36 (72.0)	63 (45.3)	47 (81.0)	99 (51.3)	**<0.001**
Poor nutritional status	31 (62.0)	32 (23.0)	29 (50.0)	70 (36.3)	**<0.001**
MNA					
Malnutrition	6 (12.0)	1 (0.7)	1 (1.7)	6 (3.1)	**<0.001**
At risk of malnutrition	25 (50.0)	31 (22.3)	28 (48.3)	64 (33.2)	
Normal nutritional status	19 (38.0)	107 (77.0)	29 (50.0)	123 (63.7)	

Notes: Quantitative variables shown as mean ± standard deviation (SD), categorical variables as number (*n*) and percentage (%). **Bold** indicates statistically significant results (*p* < 0.05). Abbreviations: BMI, Body Mass Index; PBF, Percent Body Fat; SMM, Skeletal Muscle Mass; FFM, Fat-Free Mass; ALM Index, Appendicular Lean Mass Index; SMM/W, Skeletal Muscle Mass to Weight Ratio; HGS, Hand Grip Strength; 5STS, Five Times Sit-to-Stand Test; MNA, Mini Nutritional Assessment—Long Form; BADL, Basic Activities of Daily Living; IADL, Instrumental Activities of Daily Living; SO, sarcopenic obesity.

**Table 4:** Prevalence of functional limitations in BADL/IADL by phenotype.

Variable	Body Composition Phenotypes				
	Sarcopenia	Obesity	SO	Non-Sarcopenic Non-Obese	*p* Value
	*n* (%)	*n* (%)	*n* (%)	*n* (%)	
Women	*n* = 32	*n* = 88	*n* = 27	*n* = 134	
BADL ≤ 5 IADL ≤ 26	7 (21.9)	18 (20.5)	10 (37.0)	26 (19.4)	0.237
	21 (65.6)	47 (53.4)	22 (81.5)	66 (49.3)	**0.012**
Men	*n* = 18	*n* = 51	*n* = 31	*n* = 59	
BADL ≤ 5 IADL ≤ 26	3 (16.7)	3 (5.9)	14 (45.2)	11 (18.6)	**<0.001**
	15 (83.3)	16 (31.4)	25 (80.6)	33 (55.9)	**<0.001**

Notes: Categorical variables as number (*n*) and percentage (%). **Bold** indicates statistically significant results (*p* < 0.05). Abbreviations: BADL, Basic Activities of Daily Living; IADL, Instrumental Activities of Daily Living; SO, sarcopenic obesity.

**Table 5:** Multivariate logistic regression analysis of factors associated with BADL/IADL limitations.

Variables	OR (BADL ≤ 5)	95% CI	*p* Value	OR (IADL ≤ 26)	95% CI	*p* Value
Number of chronic diseases	1.225	1.072–1.399	**0.003**	1.208	1.048–1.393	**0.009**
Number of medications	1.062	0.978–1.153	0.155	1.099	1.012–1.192	**0.024**
MNA score	0.842	0.769–0.922	**<0.001**	0.866	0.794–0.945	**0.001**
Low lower limb strength	1.591	0.877–2.888	0.127	2.261	1.202–4.253	**0.011**
Low upper limb strength	3.225	1.815–5.729	**<0.001**	4.297	2.497–7.394	**<0.001**
Low muscle mass (ALM Index)	0.817	0.359–1.860	0.630	1.063	0.525–2.153	0.866
BMI [kg/m^2^]	1.020	0.926–1.125	0.685	1.107	1.014–1.210	**0.024**
PBF [%]	1.017	0.969–1.067	0.488	0.968	0.928–1.010	0.137

Notes: **Bold** indicates statistically significant results (*p* < 0.05). Abbreviations: MNA, Mini Nutritional Assessment—Long Form; ALM Index, Appendicular Lean Mass Index; BMI, Body Mass Index; PBF, Percent Body Fat; BADL, Basic Activities of Daily Living; IADL, Instrumental Activities of Daily Living.

**Table 6:** Multivariate logistic regression models for BADL and IADL limitations by body composition phenotype (adjusted for age and sex).

**Variable**	**OR BADL ≤ 5**	**95% CI**	***p* Value**	**OR BADL ≤ 5 (Adjusted)**	**95% CI**	***p* Value**
Sarcopenia	1.054	0.48–2.300	0.895	0.716	0.308–1.667	0.439
Obesity	0.750	0.417–1.349	0.337	0.872	0.465–1.634	0.669
SO	2.976	1.579–5.608	**<0.001**	2.859	1.423–5.744	**0.003**
	**OR IADL ≤ 26**	**95% CI**	***p* Value**	**OR IADL ≤ 26 (Adjusted)**	**95% CI**	***p* Value**
Sarcopenia	2.442	1.238–4.814	**0.010**	2.037	0.959–4.328	0.064
Obesity	0.787	0.508–1.219	0.283	0.936	0.576–1.520	0.788
SO	4.057	1.985–8.290	**<0.001**	3.675	1.707–7.910	**<0.001**

Notes: **Bold** indicates statistically significant results (*p* < 0.05). Abbreviations: BADL, Basic Activities of Daily Living; IADL, Instrumental Activities of Daily Living; SO, sarcopenic obesity.

## Data Availability

All relevant data are within the manuscript and are openly available in the Zenodo repository (https://doi.org/10.5281/zenodo.16749490, accessed on 6 August 2025).
